# Neuroimmune Interactions in Fetal Alcohol Spectrum Disorders: Potential Therapeutic Targets and Intervention Strategies

**DOI:** 10.3390/cells12182323

**Published:** 2023-09-21

**Authors:** Sayani Mukherjee, Prashant Tarale, Dipak K. Sarkar

**Affiliations:** 1 The Endocrine Program, Department of Animal Sciences, Rutgers, The State University of New Jersey, New Brunswick, NJ 08901-1573, USA; sayani.mukherjee@helse-bergen.no (S.M.); pt378@sebs.rutgers.edu (P.T.); 2 Hormone Laboratory Research Group, Department of Medical Biochemistry and Pharmacology, Haukeland University Hospital, Jonas Lies vei 91B, 5021 Bergen, Norway

**Keywords:** prenatal ethanol exposure, microglia, neuroinflammation, extracellular vesicles, neuronal cell death

## Abstract

Fetal alcohol spectrum disorders (FASD) are a set of abnormalities caused by prenatal exposure to ethanol and are characterized by developmental defects in the brain that lead to various overt and non-overt physiological abnormalities. Growing evidence suggests that in utero alcohol exposure induces functional and structural abnormalities in gliogenesis and neuron–glia interactions, suggesting a possible role of glial cell pathologies in the development of FASD. However, the molecular mechanisms of neuron–glia interactions that lead to the development of FASD are not clearly understood. In this review, we discuss glial cell pathologies with a particular emphasis on microglia, primary resident immune cells in the brain. Additionally, we examine the involvement of several neuroimmune molecules released by glial cells, their signaling pathways, and epigenetic mechanisms responsible for FASD-related alteration in brain functions. Growing evidence suggests that extracellular vesicles (EVs) play a crucial role in the communication between cells via transporting bioactive cargo from one cell to the other. This review emphasizes the role of EVs in the context of neuron–glia interactions during prenatal alcohol exposure. Finally, some potential applications involving nutritional, pharmacological, cell-based, and exosome-based therapies in the treatment of FASD are discussed.

## 1. Introduction

The term fetal alcohol spectrum disorders (FASD) alludes to a group of neurodevelopmental disorders and birth defects associated with prenatal alcohol exposure (PAE). FASD includes fetal alcohol syndrome (FAS), partial FAS (pFAS), alcohol-related neurodevelopmental disorder (ARND), alcohol-related birth defects (ARBD), and neurobehavioral disorder associated with prenatal alcohol exposure (ND-PAE) [1,2]. It has been reported that over six million children worldwide are born with FASD every year [3], of which approximately 1.2 million are born with FAS. Further, the number of people with FASD between the ages of 0 and 18 years is over 11 million, and the number of people between ages 0 and 40 is more than 25 million. Individuals with FASD experience a variety of symptoms, including physical (craniofacial abnormalities), emotional, behavioral, self-regulation, communication, and learning difficulties, many of which are related to altered fetal brain development [4,5]. The brain regions that have been shown to be affected by FASD are the cerebellum, cerebral cortex, caudate nucleus, corpus callosum, and hippocampal regions [6]. The hypothalamic–pituitary axis (HPA) has also been shown to be altered by PAE, which results in lifelong instability of the brain’s stress-response system [7,8,9,10,11]. The structural and functional abnormalities of FASD have already been addressed in several reports; instead, we will critically review the results of studies dealing with neuron–glia–immune interactions, with a special focus on microglia during the pathogenesis of FASD. Additionally, we will summarize the potential treatment and management strategies for FASD.

During recent years, developmental neurobiologists have identified that the neuron–glial interaction not only contributes to neurogenesis but is also implicated in neurodevelopmental disorders [12,13]. Functionally, glial cells have been reported to support neurons, maintain homeostasis, and form myelin in the developing brain [14,15]. However, during pathological insults like PAE, the glial cell’s homeostatic function becomes altered, resulting in dysregulated neuro–glia crosstalk and neuroinflammation [16]. Within the central nervous system, glial cells comprise microglia, oligodendrocytes, astrocytes, and ependymal cells [16]. Importantly, microglia represent almost 80% of all brain-resident immune cells [17,18]. It has been reported that alcohol exposure during pregnancy affects the morphology of glial cells (including all radial glia and other transient glial structures), which induces a phenotypic shift and activates inflammatory signaling in the fetal brain. The neuroinflammatory shifts in glial cell function are detrimental to neuron survival and ultimately responsible for neuronal cell death [19,20]. Further, in developing brains, alcohol exposure perturbs neural stem cell proliferation and differentiation [21]. Therefore, to better understand FASD pathogenesis, we will emphasize neuron–glial–immune interactions in more detail. Considering that FASD is a major problem in society and no known efficient preventive measures (other than avoiding alcohol) or treatment strategies are available, early intervention, therapeutic, and management strategies must be considered to mitigate the effect of PAE and improve the life of the FASD population. This review also discusses the current state of evidence related to potential therapeutic strategies for the treatment of FASD.

## 2. Glial Cell Pathologies and Neuroimmune Crosstalk in Fetal Alcohol Spectrum Disorder

Pathophysiological FASD includes damage to multiple regions of the brain, which are interconnected to perform homeostatic brain functions. Neurogenesis, neuronal differentiation, and neuronal apoptosis are the most studied mechanisms in animal models of FASD. This review focuses on fetal alcohol effects on glial cell pathologies, including glia–neuron interactions in the context of stress-axis functions (subjects with FASD often display a stress hyper-response and anxiety behaviors [22]) and the involvement of epigenetic mechanisms in alcohol actions. The possible emerging role of sEV in glia–neuron interactions is also discussed.

### 2.1. Microglia Pathology

Microglia are usually smaller in size compared to other glial cells and have phagocytic properties, which allow them to engulf foreign particles and are considered the brain’s first line of defense [23,24]. Resting microglia have long, highly branched processes and a small cell body. In a normal physiological state, microglia (anti-inflammatory or M2) have been reported to support neurons and maintain homeostasis [25]. Several studies have shown that microglia modulate plasticity at the synapse by interacting with neurons [26,27,28], confirming their physiological function. However, microglial physiological states have been shown to be influenced by ethanol treatment, and microglia have been reported to transform into an activated physiological state or pro-inflammatory M1 type, wherein they depict an ameboid morphology, including hypertrophic cell body with short and thick processes [29,30]. These activated microglia have been shown to express more ionized calcium-binding adapter protein 1 (IBA-1), a cluster of differentiation molecule 11b (CD11b), and secrete several neurotoxic factors. It has been reported that animals fed with an ethanol-containing diet had activated microglia in the cerebellum, hippocampus, and cerebral cortex [31,32]. Other than these regions, studies from our laboratory have demonstrated that prenatal and postnatal ethanol exposures resulted in microglial activation in the hypothalamus [8,33,34,35,36,37,38,39]. These activated microglia have shown to increase the release of pro-inflammatory molecules including interleukin-1β (IL-1β), tumor necrosis factor-alpha (TNF-α), nitrite, cyclooxygenase-2 (COX-2), and inducible nitric oxide synthase (iNOS) through the activation of mitogen-activated protein kinase (MAPK) and nuclear factor-kappa B (NF-κB) signaling pathways [40], and Toll-like receptors (TLRs) [41]. The Toll-like receptor (TLR)4 is the most studied member of the TLR family that responds to inflammatory stimuli via mediating inflammatory signal transduction [42]. Interestingly, it has been reported that the effects of ethanol exposure were mediated by TLR4s present on the microglia membrane, as TLR4 knockout mice were not responsive to ethanol effects. These results imply that TLR4 and downstream signaling pathways are crucial for ethanol-induced activation of microglia [43,44]. Prenatal ethanol exposures also have been shown to elevate mitochondrial reactive oxygen species (ROS) in cortical microglia, suggesting another mechanism of immune activation in microglia [37,42,43]. Several other mechanisms have also been reported for microglia–neuron interaction in in vitro FASD models. Previous studies from our lab have shown that the conditioned media from ethanol-treated microglia contain a high level of TNF-α that induces apoptosis in cultured medio-basal hypothalamic neurons. In contrast, the adenosine 3′,5′-cyclic monophosphate (cAMP), which is considered a growth factor, has been shown to reduce TNF-α secretion by microglia and suppress the neurotoxic effects of ethanol [39]. Our laboratory further demonstrated that ethanol induces apoptosis in cultured hypothalamic neurons primarily through an increase in ROS and a decrease in antioxidant levels. During the developmental period, brain-derived neurotrophic factor (BDNF) promotes neuronal growth and plasticity by activating tyrosine kinase B (TrkB) and associated downstream signaling pathways, while cAMP regulates cellular oxidative stress [35]. We have shown that ethanol reduces the levels of cAMP and brain-derived neurotrophic factor (BDNF) in cultured neurons that, eventually trigger neuronal apoptosis [35,37,39].

Microglial functions are also regulated through various neurotransmitter receptors. We have found that ethanol alters the signaling of mu-opioid receptors (MOR) and delta-opioid receptors (DOR) in microglia. This was revealed by the findings that MOR agonist [D-Ala 2, N-MePhe 4, Gly-ol]-enkephalin (DAMGO) elevated the release of pro-inflammatory cell signaling proteins, while a DOR agonist [D-Pen2,5] enkephalin (DPDPE) increased the secretion of anti-inflammatory cytokines and blocked the ability of ethanol to induce microglial pro-inflammatory actions. In neonatal rat pups, alcohol feeding increased the levels of microglial MOR protein and pro-inflammatory signaling molecules in the hypothalamus, while naltrexone (non-specific MOR blocker) blocked the effects of alcohol. Additionally, activation of MOR or DOR counteracted each other’s effects on hypothalamic neurons [38].

Monocyte chemoattractant protein-1/chemokine receptor 2 (MCP-1/CCR2) signaling has also been shown to be involved in ethanol-induced neurotoxicity involving the microglia–neuron crosstalk [45,46,47]. Postnatal ethanol exposure in animal models of FASD has been shown to trigger microglial activation with the elevated secretion of MCP-1, which activates the MCP-1/CCR2 signaling pathway in neurons of the cerebellum and cortex, causing neuronal death. These effects were abolished in MCP-1 and CCR2 knockout mice or in mice treated with the MCP-1 synthesis inhibitor Bindarit or the CCR2 antagonist RS504393, suggesting that MCP-1/CCR2 chemokine signaling plays an important role in ethanol-induced microglial activation, neuroinflammation, and neuro-apoptosis [48].

**Figure 1:**
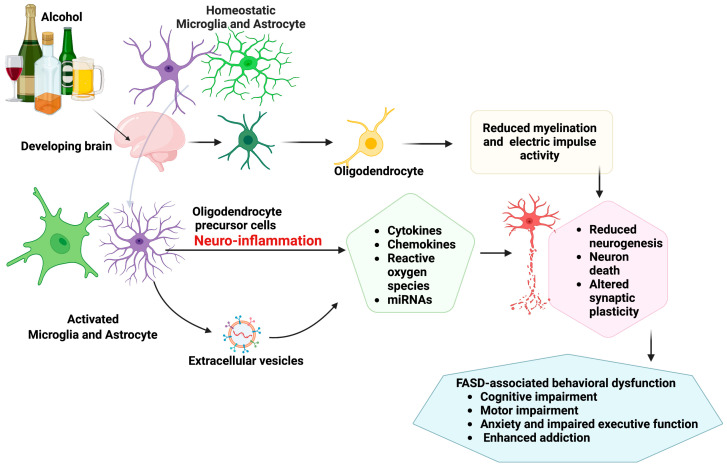
A schematic diagram showing ethanol-induced alterations in glial cell functions. Early ethanol exposure during development causes a shift from resting microglia and astrocytes to activated microglia and astrocyte phenotypes. These inflammatory glial phenotypes secrete extracellular vesicles containing inflammatory molecules, including cytokines, chemokines, and miRNAs, which are detrimental to neurons associated with regulation of stress-axis function. Ethanol exposure also alters oligodendrocyte functions, which has long-term effects on neuronal myelination, affecting synaptic plasticity. These events may result in FASD-associated cognitive, behavioral, and motor impairments.

Together, these studies show that alcohol exposure activates microglia, which then release toxic factors that promote neuronal apoptosis (Figure 1). Nevertheless, neuron–glia–immune interactions are extremely complex, and microglia are crucial but not the only glial cells that are involved in neuroimmune interactions. Further down, we will discuss the roles of other glial cells that participate in neuroimmune interactions.

### 2.2. Astrocytes Pathology

In the early developing brain, astrocytes originate from a radial glial cell population surrounding the ventricular zone, like neurons and oligodendrocytes [49,50]. During the development of CNS, astrocytes have been shown to perform an integral role in trophic, structural, and metabolic functions [50,51]. Glial fibrillary acidic protein (GFAP), the intermediate filament-III protein and a key structural element expressed in mature astrocytes and developing astrocytes, is one of the most commonly used astrocyte markers [52]. However, astrocytes may become hyperactive (A1 astrocytes) and undergo dramatic morphological, molecular, and functional changes in certain circumstances such as inflammation, neurodegenerative disease, neurodevelopmental disease (including FASD) [53,54], and acute injury [55,56]. These A1 reactive astrocytes produce pro-inflammatory substances and neurotoxins that can cause neuronal death and disrupt brain homeostasis. According to recent reports, complement classical cascade component C3 is a powerful marker for A1 (only in neurodegenerative diseases or during brain injury), unlike anti-inflammatory A2 and resting astrocytes [57,58]. Astrocytes secrete several crucial protein molecules that are involved in neuronal development, such as neurite outgrowth and synaptogenesis [59]. Several studies have identified the involvement of astrocytes in the prenatal ethanol-altered development of surrounding neurons in the brain [60,61,62,63]. The interaction between glial cells (including astrocytes) and vasculature is critical for the maintenance of BBB and the proper functions of the nervous system. Recent studies suggest that ethanol may trigger a dysfunctional phenotype in brain endothelial cells, leading to impairment of cortical vascular network formation and endothelial cell-induced abnormalities in astrocyte functions that could affect BBB establishment in the developing brain [63]. Thus, it is important to understand how astrocyte functions are altered during alcohol exposure to determine the exact neuron–glia–immune relationship.

In cultured astrocytes, ethanol promotes the activation of pro-inflammatory interleukin 1 receptor (IL-1R)-associated kinase, extracellular signal-regulated protein kinase 1/2 (ERK1/2), p38, and Jun N-terminal kinases (JNK) and the production of ROS [64,65,66,67]. Activated pro-inflammatory signaling pathways then increase the production of pro-inflammatory molecules such as IL-1β, iNOS, and COX2 in cultured astrocytes [53,57]. A study has shown that ethanol-activated astrocytes stimulate microglia, which produce inflammatory mediators in the brain that may contribute to FASD pathology [57]. Inflammasomes are an important component of the innate immune system and nucleotide-binding protein. Leucin-rich containing family pyrin-domain-containing 3 (NLRP3) is the most studied and well-characterized inflammasome. Aberrant activation of NLRP3 has been linked to various degenerative diseases [68]. The activation of NLRP3 in astrocytes by ethanol has been found to cause inflammation and neuronal death [44,69,70].

Together, these results indicate that ethanol exposures trigger pro-inflammatory signaling pathways in astrocytes and that the bidirectional communications between astrocytes and microglia further modulate CNS inflammation through the release of multiple cytokines and inflammatory mediators.

### 2.3. Oligodendrocyte Pathology

The oligodendrocyte is the myelinating cell of the CNS [60]. In addition to producing and maintaining myelin, oligodendrocytes maintain the structure and provide protection to unsheathed axons. Oligodendrocyte precursor cells (OPCs) are produced around embryonic day (ED)16 in rats and gestational age (GA) 5.5 weeks in humans. Maturation and myelination begin during the second trimester, around 20 weeks of gestational age in humans, and continue postnatally [71]. Therefore, first-trimester exposure to ethanol is most likely to affect OPC function and differentiation [72]. A case–control study performed in 20 alcohol-exposed fetuses from elective pregnancy terminations revealed cytokine dysregulation (TNFα, MCP-1). Growth-regulated protein alpha (GROα) that inhibits migration of oligodendrocyte precursors was upregulated, while neuroprotective cytokine insulin-like growth factor-1 (IGF-1) was downregulated [72]. In a mice model of FASD, ethanol treatment from PD4–9 was found to diminish the expression of the myelin proteolipid protein (PLP) gene, PLP1, associated with mature oligodendrocytes, along with several genes expressed in OPCs [72]. In summary, developmental exposures to ethanol are associated with delayed maturation of oligodendrocytes that may cause a long-lasting effect on myelination in children and adolescents with FASD.

### 2.4. Neuron–Microglia Immune Interactions and Epigenetic Involvement in Pathophysiology of FASD

**Figure 2:**
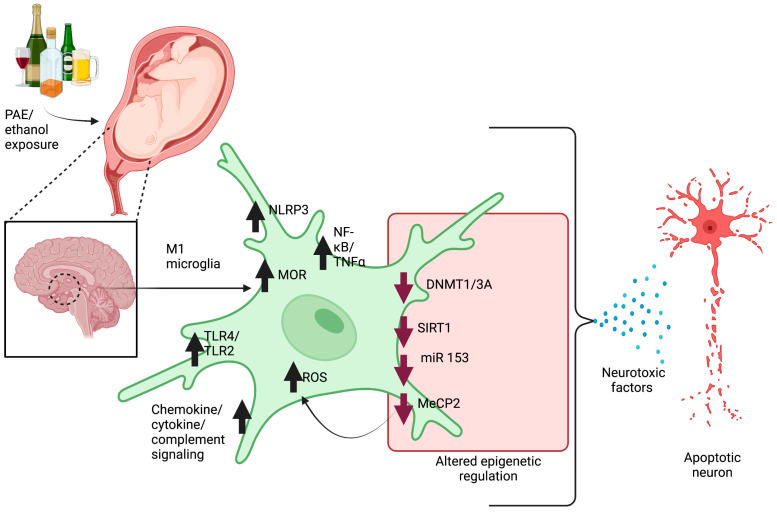
A schematic diagram showing ethanol-induced changes in microglial cell functions. Early ethanol exposure during the developmental period causes phenotypic shift from resting microglia to activated microglia (M1). The most common signaling pathways that are upregulated during alcohol-induced microglial priming are TLR2/4, chemokine, cytokine, complement, MOR, NLRP3, NF-kB/TNF-α, and ROS signaling. The most common epigenetic alterations include decrease in DNMT1/3A, SIRT1, miR153, and MeCP2. All these altered signaling mechanisms trigger the release of neurotoxic factors from primed microglia and cause apoptotic death of neurons.

Epigenetics entails the study of reversible genetic changes that are independent of changes in the DNA sequence. The gene-regulatory epigenetic changes involve DNA methylation, histone modification, and non-coding RNAs such as microRNA expressions [73,74,75,76]. Epigenetics has been recognized to play an important role in the emergence of a specific phenotype (M1 or M2) of microglia following an environmental challenge [73]. For example, upon stimulation with lipopolysaccharide (LPS), histone methyltransferase activity is increased, leading to an increase in tri-methylation of histone H3 lysine 27 (H3K27) and pro-inflammatory gene nuclear factor-κB (Nfkb1) levels in mouse microglia [64,65]. Also, the activation of TLR4 signaling by LPS is shown to increase tet methylcytosine dioxygenase 2 (TET2) levels and stimulate the expression of pro-inflammatory cytokines in mouse microglia [66]. TET2 has been shown to facilitate the oxidative conversion of 5-methylcytosine (5 mC) to 5-hydroxymetylcytosine (5 hmC) [77]. It should be noted that the expression of epigenetic modifier genes Mecp2, Tet2, Dnmt1, and Dnmt3a are altered in a variety of rodent models of FASD [8,78,79,80,81,82,83,84]. MicroRNAs (miRNAs or miRs) are non-coding small single-stranded RNA containing 21–23 nucleotides. MiRNA post-transcriptionally regulates gene expression via binding to 3′-UTR of target mRNA and repressing its translation [85,86]. The expression of miR153 has also been shown to be decreased in mouse fetal cerebral cortical-derived neural progenitor cells exposed to ethanol [71]. Also, the addition of an miR153 mimic to mouse microglia has been shown to reduce the release of TNF-α [72]. Similar effects have been reported in zebrafish in which exposure to ethanol from 4 to 24 h post-fertilization decreased the expression of miR153c, a zebrafish homolog of miR153 [73]. Further, silencing of miR153c triggered the phenotypes like those of zebrafish subjected to ethanol from 4 to 24 post-fertilization, suggesting a distinct ethanol-induced change in miRNA expression both in zebrafish and rodents. Thus, it could be predicted that the epigenetic dysregulation caused by exposure to alcohol during development may induce neuroinflammation through polarization of microglia into pro-inflammatory phenotypes (Figure 2).

An epigenetic mechanism may also participate in the development of microglial hyper-response [87,88]. We have recently shown that adult rats with neonatal alcohol pre-exposure showed an exaggerated peripheral stress hormonal response to LPS due to a hyperactive microglia response involving Cd11b activation, TNF-α expression, and IL-6 production. Interestingly, blocking microglia activation with minocycline treatment during alcohol exposure reduced the microglial sensitivity to LPS in adult PAE rodent animals. Hyperactive microglia response to LPS in PAE animals was associated with increased histone H3 acetyl lysine 9 (H3K9ac) enrichment at TNF-α and IL-6 promoter regions, suggesting a possible epigenetic mechanism for the long-term immune disruption due to hypothalamic microglial priming [33].

It is evident from the data of these studies that alcohol-induced epigenetic abnormalities can alter microglial activity to make them more neurotoxic. Further research may be necessary to understand how other glial cells are epigenetically primed and interact with neurons during the pathogenesis of FASD, allowing for the development of novel therapies to combat stress-related problems associated with FASD.

### 2.5. Extracellular Vesicles in Neuron–Glial Crosstalk

Extracellular vesicles (EVs) are membranous structures derived from the endosomal system. They are present in biological fluids and are recognized as an alternative mechanism for intercellular communication, as they allow the exchange of proteins, lipids, and genetic information between cells. Several recent studies have demonstrated that EVs released by alcohol-activated glial cells can harm nearby neurons by the transfer of neurotoxic factors. The purpose of this section is to review recent advances in EV-mediated neuron–glia interactions during alcohol exposure.

We have recently shown that exposure to ethanol during the developmental period dysregulates the normal communication between microglia and POMC neurons for maintaining homeostasis by releasing apoptotic factors that involve complement proteins C1q, membrane attack complex (MAC), and reactive super-oxygen species (ROS), thereby causing POMC neuronal death [34]. As discussed earlier, POMC neurons known to regulate stress functions are reported to be killed by developmental alcohol exposure due to the activation of microglial immune cells in the brain. Briefly, in both in vivo and in vitro models, we found that ethanol-treated microglial exosomes (30–150 nm) show higher numbers and increased ability to kill POMC neuronal populations. Proteomic analyses of exosomes from cultured microglial cells revealed that ethanol treatment upregulated many proteins, including several complement factors. Further, we have found that ethanol treatment elevated the deposition of the complement protein C1q on β-endorphin neuronal cells in both in vitro and in vivo models. We further demonstrated that C1q blockers prevented the death of β-endorphin neurons by reducing the deposition of complement factors C3a/b, C4, and or by blocking MAC/C5b9 formations [34]. These data suggest that exosomes play an important role in microglia–neuron interaction. However, the question that remains to be determined is whether EV-mediated inflammation is directional. A study by Crews et al. found that alcohol treatment in organotypic brain slice (OBSC) cultures altered microvesicle (MV) cargo and induced a unique immune gene signature in microglia [89]. These authors found that MV-treated microglia showed an increase in TNF-α, IL-1β, purinergic 2 receptor Y12 (P2RY12), CX3C motif chemokine receptor 1 (CX3CR1), and microglial presynaptic gene C1q while showing a decrease in homeostatic gene type1 transmembrane protein 119 (Tmem119) and the phagocytic gene triggering receptor expressed on myeloid cells 2 (TREM2). On the contrary, microglia depletion prevented MVs’ pro-inflammatory activity—demonstrating that MVs from the brain microenvironment can activate microglia and that MVs mediate inflammation in a complex manner.

Similar to microglia, ethanol has been shown to promote the EVs secretion from astrocytes by inducing the pro-inflammatory signaling pathways, including TLR4, NLRP3, IL-1R, NF-κB, and caspase-1 (apoptotic marker) [67,90]. Several studies have found that astrocyte-derived EVs were captured by cortical neurons, which results in neuronal death due to an increase in ROS production and the expression of inflammation-related proteins and miRNAs [90,91,92]. These data suggest that neurons and astrocytes may communicate with each other, and ethanol may adversely affect this communication.

It is also important to know how ethanol exposure directly affects fetal neuronal stem-cell-derived EVs [93,94,95]. Most neurons in the adult brain are generated by neural stem cells (NSCs) during the first and second trimesters of pregnancy. It has been shown that alcohol exposure during prenatal development results in compromised brain growth during this crucial neurogenic period due to altered NSC expression of major neurogenic miRNAs [95]. Interestingly, miRNA cargo is trafficked between cells by EVs in the NSC microenvironment. The altered miRNA contents by ethanol exposure result in aberrant neural progenitor growth and maturation [92]. These data suggest that EVs can circulate bidirectionally between neurons and glia within the brain and play an important role in the development of FASD by prenatal ethanol.

## 3. Therapeutic Intervention Strategies for Fetal Alcohol Spectrum Disorders (FASD)

**Figure 3:**
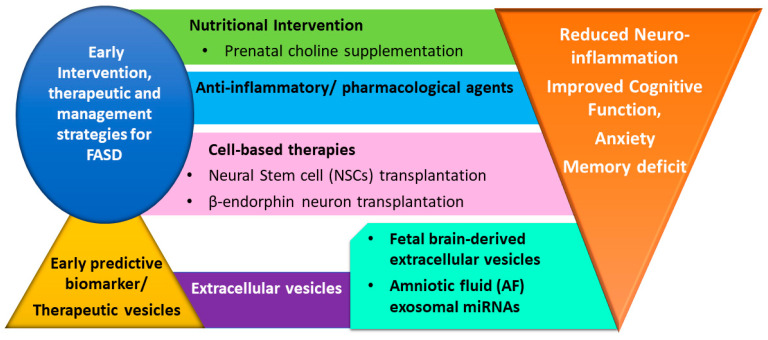
A schematic diagram showing the early intervention, therapeutic, and management strategies to improve the cognitive function in FASD. Extracellular vesicles may be considered as potential biomarkers as well as therapeutic vesicles for FASD.

Structural changes in the brain are responsible for the learning and behavioral deficit observed in children born with FADS, which they continue to experience throughout their lives. Cognitive development is crucial for the social, recreational, and academic participation of children. However, awareness of this fact is not a cure for a woman who learns about their pregnancy and stops alcohol consumption during their first trimester. Indeed, alcohol-induced damage has already been done [96]. There is no absolute care to treat the negative effects of FASD. The early intervention and treatment strategies can be helpful to improve cognitive and social abilities [97]. The discussed measures below showed some potential to improve cognitive function in the FASD population (Figure 3).

### 3.1. Nutritional Supplementation

FASD is a lifelong condition with neuroimmune, cognitive, and behavioral dysfunction. Therefore, early nutritional intervention might provide benefits for neurodevelopmental deficits. Choline is an essential micronutrient for neurological development and brain function [98,99]. In a long-term study, FASD children with 2.5–5 years were followed for approximately 7 years after the initial efficacy trial of choline supplementation. Follow-up MRI scans and executive function tests with these children receiving choline supplementation showed better performance with several low-order executive functions (e.g., processing speed) and higher white matter microstructure organization in the splenium of the corpus callosum as compared to those in the placebo group [100,101]. In an animal study, Sprague–Dawley rat pups received ethanol (5.25 g/kg/day) from PD 4–9 and choline chloride (100 mg/kg/day) from PD 10–30—the hippocampus collected from these rats at PD 35 or PD 60 showed choline mitigated the long tasting effect of ethanol on inflammatory tone via modulating the ratio of pro-to-anti-inflammatory cytokines [102,103]. In vitro electrophysiology experiments at PD 30–35 in choline-supplemented juvenile males and females from PD 10–30 showed a positive effect on hippocampal synaptic physiology that may be attributed to choline-related improvement in cognitive function [104]. Prenatal supplementation of choline (642 mg/L) in mice was found to prevent gross developmental abnormalities associated with prenatal alcohol (25%) exposure [105]. Additionally, choline supplementation prevented prenatal alcohol-induced alteration in *RZRβ* and *Id2* genes—implicated in pattering on the neocortex along with rescuing sensorimotor behavior dysfunction [105].

### 3.2. Anti-Inflammatory and Pharmacological Agents

Several animal models of FASD have identified neuroinflammation as a hallmark of FASD-associated neuropathological events [69]. Therefore, anti-inflammatory agents might be considered as a potential protective therapeutic option for FASD. Peroxisome proliferator-activated receptors (PPARs) belong to the nuclear family of proteins. PPAR-γ agonists have been studied to suppress the production of the IL-12 family of cytokines in in vitro cultured microglia and astrocytes [106]. In vivo administration of PPAR-γ agonist 15-deoxy-Δ12,14 prostaglandin J2 has also been found to be protective for Purkinje cell neurons via suppressing microglia activation in a postnatal animal model of PAE [107]. Postnatal ethanol exposure has been shown to reduce ω-3 polyunsaturated fatty acid docosahexaenoic acid (DHA) in the developing brain, which is critical for synaptic plasticity and neuronal development [108]. Thus, DHA, which is another PPAR-γ agonist, has been used in baby formula and dietary supplements during pregnancy [109]. Postnatal DHA (10 g/kg in artificial milk diet) supplementation in rats between PD11 and PD20 has been found to ameliorate alcohol-induced behavioral deficit, suggesting the therapeutic potential of FASD [110]. However, the neuroinflammation suppressive effect of PPAR-γ agonist still needs to be evaluated in in vivo models of FASD. The anti-inflammatory agent minocycline has been evaluated to suppress microglial activation via blocking expression of pro-inflammatory cytokines IL-6, MCP-1, CCR2, and GSK3β and protecting neurodegeneration in a postnatal rat model of FASD [33,38,48,111]. Prenatal and lactational alcohol-exposed mice models were used to investigate the preventive effects of curcumin on cognitive impairments. Male mice treated with curcumin during the peri-adolescence period (PD 28–35) showed improvement in anxiety and memory deficits when evaluated for behavior in adulthood (PD60) [112]. Another anti-inflammatory agent, cannabidiol (CBD), was also reported to ameliorate cognitive deficits in the PAE mice model and restore elevated levels of TNFα and IL-6 in the hippocampus, thus suppressing ethanol-induced neuroinflammation [113]. Prenatal treatment with epigallocatechin-3-gallate in a mice model of FASD was shown to rescue fetal growth restriction and suppressed alcohol-induced changes in placental angiogenic factors while partially ameliorated neuronal nuclear antigen (NeuN), (doublecortin) DCX, and GFAP levels [114].

Metformin is an approved first-line treatment drug for diabetes that has been studied for its anti-inflammatory, antioxidant, and anti-apoptotic activity in animal models of FASD. Metformin treatment was found to suppress ethanol-induced neuroinflammation and hippocampal apoptotic death of neurons in adult male rats [115]. Inter-neuronopathy has been identified to contribute significantly to the patho-etiology of FASD; specifically, in utero ethanol exposure was found to potentiate the depolarizing activity of gamma-aminobutyric acid (GABA) in GABAergic cortical interneurons in developing embryonic brain [116]. The chloride importing Na^+^-K^+^-2Cl^−^ isoform one cotransporter (NKCC1) plays a crucial role in GABA-activated responses. Bumetanide, an antagonist of NKCC1 cotransporter, has been found to mitigate the ethanol-induced inter-neuronopathy in the prefrontal cortex and associated behavioral deficit in mice models of FASD [116]. These findings identify the potential utility of NKCC1 (sodium/potassium/chloride cotransporter) as a pharmacological target for early intervention and management of FASD. Calcium-activated potassium channel Kcnn2 elevated expression in motor cortex neurons is implicated in deficit in motor learning skills. Postnatal administration of Kcnn2 blocker tamapin (100 nM) in mice model of FASD was reported to improve motor learning impairments [117]. Collectively, these studies suggest the potential of anti-inflammatory agents in ameliorating ethanol-induced neuroinflammation with the possibility of using it as a treatment for FASD. However, extensive animal and clinical research is required to determine the dose, therapeutic window, and mechanistic insight for the effects of these anti-inflammatory agents.

### 3.3. Cell-Based Therapies

Clinically, FASD is attributed to the loss of neurons or neuronal function. Thus, neural stem cells (NSCs) may be considered as a possible treatment for FASD [118]. Intravenous transplantation of NSCs in rat models of PAE have been found to recover rats from alcohol-induced brain damage to neural network and cognitive function [119]. Moreover, the transplanted NSCs have been found to migrate wide areas of the brain and mitigate behavioral abnormalities in rat models [120]. Children with FASD are also prone to various urinary and respiratory tract infections attributable to compromised natural killer (NK) cell activity [121]. NK cells activity can be regulated by the neuroendocrine system, which involves hypothalamic β-endorphin neurons [122]. β-endorphin neurons are reduced in adult PAE rats. Therefore, previous studies from our laboratory explored the possibility of transplantation of β-endorphin neurons in reducing the stress response as well as altered immune functions in fetal exposed rats [22,123]. Studies from our laboratory showed that in vitro differentiated β-endorphin neurons transplanted to the hypothalamus of ethanol-exposed offspring produced β-endorphin-precursor peptide proopiomelanocortin (POMC), reduced corticotropin-releasing hormone (CRH) neuronal response to immune stress, and increased the cytolytic activity of NK cells [123,124,125]. These results are indicative of retaining the biological functionality of transplanted β-endorphin neurons [126]. However, there are still uncertainties about how transplanted NSCs or β-endorphin neurons exert their neuroprotective effects. It is speculated that these transplanted neurons may integrate with host tissue to promote endogenous neurogenesis or via secreting various trophic factors [127]. Additional, future mechanistic investigation is required prior to the clinical implementation of NSCs for cell therapy for FASD.

### 3.4. Small Extracellular Vesicles (Exosomes) as Predictive Biomarker and Therapeutic Carrier Vesicles

Brain-derived exosomes (30–150 nm) have been studied in relation to ethanol exposure and may be considered novel biomarkers to diagnose early FASD in fetuses [128,129]. For example, fetal brain-derived exosomes were obtained from the maternal blood of 10 mothers who consumed alcohol. Several brain-derived exosome markers were studied in correlation with eye size in all 10 alcohol-exposed fetuses and their age-matched controls. A strong correlation has been established between myelin basic protein (MBP) and eye diameter, which might be considered as strong predictive biomarkers for the development of FASD [130]. Exosome-derived RNA content was also studied in the amniotic fluid (AF) in the rat fetal alcohol (FAE) exposure model. RNAseq analysis identified several AF-exosome-miRNAs that were altered in response to maternal ethanol exposure. Significant dysregulation was observed in miRNAs (miR-199a-3p, miR-214-3p, and let-7g) regulating osteogenic differentiation in rat bone marrow stem cells [131]. Another study using the sheep model of prenatal alcohol exposure suggests maternal–fetal miRNA transfer wherein plasma circulatory miRNAs including miR-9, miR-15b, miR-19b, and miR-20a were found specifically altered in both alcohol-fed pregnant ewe and newborn lamb [132]. These studies signify the in utero exosomal transfer of ethanol effect on stem cell maintenance and differentiation, including miRNA profile. It would be interesting to study if correcting the altered level of exosome-derived miRNAs in amniotic fluid using miRNA mimics or inhibitors can mitigate the detrimental effect of alcohol on development. Also, altered exosome-miRNAs may be considered as early predictive biomarkers for the development of FASD.

### 3.5. Outstanding Questions

(i) Early EV-based intervention strategies have the potential to mitigate FASD-associated neurobehavioral abnormalities and need to be further investigated;
(ii) Early diagnosis of FASD using exosome-derived proteins or miRNAs as predictive biomarkers will be helpful in devising early intervention strategies.

## 4. Summary

The development of FASD may involve complex interactions between CNS cells in the fetal brain upon in utero alcohol exposure. Growing evidence suggests that the altered activity of glial cells is one of the reasons for neuronal death during PAE. In this review, we emphasized the alcohol-induced molecular and phenotypic changes in glial cells. We discussed several mechanistic pathways that trigger the release of neurotoxic factors from microglia. Besides microglia, we have discussed the roles of astrocytes and oligodendrocytes in neuronal cell function and synaptic activity. The emerging role of exosomes has also been emphasized here. We discussed microglia-derived exosomes, which are released upon ethanol exposure and found to contain apoptosis-inducing factors responsible for the death of β-endorphin neurons, regulating body stress responses. We also showed astroglia and neuron-derived exosomes play important roles in the etiology of FASD. However, there are yet unexplored areas in glia–neuron interactions that need to be addressed in the context of FASD.

Individuals with FASD can benefit from early intervention and management to improve their cognitive function and quality of life. We have documented several explorative therapeutic approaches for FASD. Nutritional supplementation, specifically choline, has been suggested to have protective effects, while anti-inflammatory agents are shown to reduce neuroinflammation and, thus, provide neuro-protection in animal models of FASD. However, the clinical implication of these intervention strategies needs to be determined. Cell-based therapies with transplantation of NSCs and in vitro differentiated β-endorphin neurons have been found to improve the stress response in FASD, but understanding the mechanisms for cell transplantation needs further research. Early diagnosis of FASD might be helpful to devise effective preventive strategies to minimize abnormalities associated with FASD. Fetal-derived exosomes from maternal blood or amniotic fluid might be considered a source of predictive biomarkers (protein, miRNAs, etc.) for FASD.

## Data Availability

Not applicable.
